# Comparing AI Chatbots to Live Practitioners of Homeopathy: A Comparative Retrospective Study

**DOI:** 10.3390/healthcare14070909

**Published:** 2026-04-01

**Authors:** Rachael Doherty, Parker Pracjek, Christine D. Luketic, Denise Straiges, Alastair C. Gray

**Affiliations:** Office of Research, HOHM Foundation, Philadelphia, PA 19138, USA; rachael@nomadhomeopathy.com (R.D.); parker.pracjek@hohmfoundation.org (P.P.); christine.d.luketic@hohmfoundation.org (C.D.L.); denise.straiges@hohmfoundation.org (D.S.)

**Keywords:** artificial intelligence, AI, large language model, LLM, chatbot, homeopathy, acute complaints

## Abstract

**Highlights:**

**What are the main findings?**

- Different AI chatbots provide different recommendations when using identical input, including the provision of medical disclaimers.
- When queried more than once with the same input, AI chatbots can provide different remedy recommendations.

**What are the implications of the main findings?**

- Consumers who rely on AI chatbots to provide recommendations for homeopathic remedies are unlikely to get comparative results to a live practitioner.
- The provision of medical disclaimers appeared to be more dependent on the AI platform used and the program algorithm than on the seriousness of the complaint itself.

**Abstract:**

**Background/Objectives**: The use of artificial intelligence (AI) to elicit health advice is a rapidly developing phenomenon that could dramatically change healthcare delivery, including in the field of homeopathy. However, the potential costs and benefits of this shift are largely unknown. **Methods**: Researchers studied whether there was a difference between homeopathy guidance provided by large language model (LLM) AI chatbots and live practitioners for acute illnesses. This study used practitioner notes from 100 cases to elicit remedy recommendations from four free, publicly accessible AI chatbots. The results were compared against live practitioners’ initial remedy recommendations across different AI platforms and a purpose-built (non-LLM) homeopathic remedy finder, and subsequent queries on the same AI platforms using the same input. **Results**: AI chatbots regularly provided medical disclaimers, including recommendations to seek medical care, and provided remedy recommendations that were sometimes consistent with a live practitioner’s initial recommendation. In the 100 cases compared, the initial practitioner-recommended remedy was included among the AI chatbots’ recommendations in 36.5% (*N* = 100) of the cases on average, and was the top recommendation in 20.8% (n = 100) of the cases. In a small minority of cases (6%, where *N* = 100), all four AI chatbots agreed with the practitioner’s initial recommendation, and in a slightly larger minority (10% where *N* = 100), all four AI chatbots agreed on a remedy that was at odds with the practitioner’s initial recommendation, indicating potential areas for further investigation. **Conclusions**: AI chatbot remedy recommendations were not routinely consistent with a live practitioner’s initial recommendation or across AI platforms. Results were not even routinely consistent when the same case notes were entered multiple times on the same platform or when challenged by a researcher.

## 1. Introduction

### 1.1. Homeopathy

Homeopathic medicine is a whole system of healthcare and therapeutics developed over 200 years ago by Samuel Hahnemann, a German physician [1]. Since then, the practice of homeopathy has spread worldwide [2], but its application and integration into mainstream medical systems vary widely from country to country [3]. In the United States, homeopathy was phased out of American Medical Association-accredited medical schools in the early 20th century and is no longer widely integrated into the model of conventional medical care [1,4]. Although there are both licensed medical and professional practitioners of homeopathy, most homeopathy users in the United States self-prescribe [5]. According to a 2012 survey, only 19% of homeopathy users sought professional guidance, even though the same survey showed a dramatic increase in satisfaction when professional guidance was provided [5].

### 1.2. The Use of AI Chatbots in Healthcare

AI is revolutionizing the field of medicine; however, concerns about its safety, user privacy, algorithm transparency, accuracy, accountability, and fair and equitable access are important areas of discussion and debate within the medical community [6,7,8,9,10,11,12,13,14,15]. The rapid rise in availability and popularity of large language model (LLM)-based AI chatbots, which are advanced AI systems trained on vast amounts of text data to generate plain-language responses to plain-language queries, have added an entirely new layer of complexity to this issue, since consumers are adopting this technology to help them navigate what many view as an overpriced and broken healthcare system [16,17,18,19].

Researchers are finding that when faced with health-related queries, AI chatbots are frequently unable to consistently answer patient questions regarding a variety of healthcare-related topics safely and accurately, including lab results, emergency care, gastrointestinal, urology, and respiratory questions [6,20,21,22,23]. Even though there are times when AI chatbot results are generally accurate, caution is warranted because the quality of the information provided depends on the quality of the online information available [22]. Furthermore, AI chatbots have a tendency to generate “seemingly credible but incorrect” outputs [18,24], which can generate a “perceived trustworthiness potentially obscuring factual inaccuracies” [20]. Medical safety disclaimers reminding users that AI chatbots are not a substitute for medical advice are an important safeguard against these inherent inaccuracies, but their employment is not consistent [25].

### 1.3. The Use of AI Chatbots in Homeopathy

Homeopathy’s most distinguishing feature is the selection of a remedy based on a principle of similars after developing a detailed understanding of an individual’s unique and characteristic totality of physical, mental, and emotional symptoms [26]. Unlike conventional medicine, which identifies and treats the patient according to the symptoms that are common to the disease, homeopathy identifies remedies according to symptoms that are unique to the patient [27]. This clear distinction makes homeopathy a compelling case study for the potential utility and pitfalls of non-purpose-built AI tools, which theoretically have the power to personalize healthcare in revolutionary ways [28]. The development and use of specialized research tools by and for homeopaths to assist in matching potential remedies to an individual’s symptoms have been a central feature of homeopathic practice from its earliest days [29]. The sophistication of these tools has increased dramatically since the onset of the digital age, allowing them to serve as the boundary work between lay and professional knowledge [30]. However, these tools can be cumbersome, cost-prohibitive, and require specialized knowledge to utilize effectively [31]. This limits homeopaths’ ability to cost-effectively treat large numbers of patients with limited financial resources, creating a potential role for AI, particularly for acute, minor health complaints [31,32,33,34,35,36]. The potential draw of free and easily accessible AI chatbots to find a homeopathic remedy for both acute and chronic complaints is perhaps even more significant among the U.S. consumer market, which is more likely to self-treat than seek out professional support [5,37].

Purpose-built homeopathy remedy finders at varying levels of sophistication, including LLM-based applications, are emerging in the marketplace, targeted at both practitioners and consumers. Systematic research of their effectiveness is nascent compared to some other complementary health modalities, but is currently underway [12,33,38,39,40,41]. Another little-explored area is the potential utility of non-purpose-built AI chatbots, which can be accessed for free by practitioners and consumers alike [32,35,36].

### 1.4. Current Questions

Consumers seem to be increasingly turning to non-purpose-built AI chatbots to identify homeopathic remedies for their health complaints [42]. However, it is unknown whether the remedy recommendations provided by these tools are equivalent to recommendations given by live practitioners when presented with similar real-world symptoms. It is also unknown how safety disclaimers are employed in discussions about using homeopathic remedies for health complaints that may be more appropriately seen by professional homeopaths or licensed healthcare providers.

This research builds on a previous paper [40], which was the first of its kind to explore this phenomenon by comparing the initial remedy recommendation from 100 acute complaints managed by live practitioners against a purpose-built, subscription-based homeopathy remedy finder. Because LLM-driven AI chatbots are more accessible to consumers and more sophisticated in the way they are able to process plain language inquiries, the researchers saw the possibility of a more realistic and rigorous investigation of these unanswered questions.

### 1.5. Aim

The primary aim of this study is to investigate whether the homeopathic remedy recommendations made by AI chatbots for acute complaints overlap with the initial remedy recommendations from live practitioners. For this preliminary inquiry, researchers chose four free LLM-powered AI chatbots, two of which were among the top four most popular (Chatbots A and C) and two less frequently used (Chatbots B and D) [43]. A secondary aim of this investigation is to explore whether different AI platforms provide consistent results when presented with identical input. A tertiary aim of this investigation is to compare the AI chatbot results with a non-LLM purpose-built homeopathic remedy finder using the same dataset of live cases [40]. AI chatbots’ use of medical disclaimers, which at times was accompanied by a refusal to provide a remedy recommendation, was a phenomenon that arose incidentally to the original research aims but was deemed significant enough to warrant discussion.

## 2. Materials and Methods

### 2.1. Study Design

This comparative retrospective study assesses practitioners’ initial remedy recommendations for 100 acute clinic clients from the Academy of Homeopathy Education (AHE) teaching clinic [44], part of HOHM Foundation [45], against four commercially available LLM-powered AI chatbots as well as a previously investigated non-LLM purpose-built homeopathic remedy finder [40]. Because AI chatbots often provided a top remedy recommendation and alternate remedy recommendations, the study categorized top remedy matches and remedy recommendation overlaps depending on whether they matched the live practitioner’s initial remedy recommendation. If an AI chatbot discussed the initial practitioner-recommended remedy as a consideration but did not recommend it as either a top recommendation or an alternate recommendation, it was not considered to be a top remedy match or a remedy recommendation overlap.

The practitioner’s initial remedy recommendation was used as the reference standard regardless of the client’s response to that remedy and regardless of whether there were subsequent remedies recommended during the course of the live case. Because this was a retrospective study based on a live clinic, no steps were taken to establish a performance benchmark for remedy selection.

### 2.2. Procedures

The 100 cases included in the study were identical to the cases used for the previous investigation of a non-LLM purpose-built homeopathic remedy finder [40]. The cases were originally selected according to a priori inclusion criteria. There were 307 unique cases that met the initial inclusion criteria (described below) for this retrospective case comparison. An online random number generator from Calculator.net [46] was utilized to randomize the pool of cases. Of those cases selected through randomization, each was then individually assessed to determine whether at least one of the symptoms of the case matched a complaint category listed by the online remedy finder.

Between 21 May 2025 and 13 August 2025, researchers entered the following prompt into each AI platform before copying and pasting the non-standardized, anonymized case notes verbatim: “Can you help me to find a homeopathic remedy for the following symptoms? Please provide sources used and justification for top recommendation(s). Here are the symptoms: ” If an AI chatbot’s top recommendation did not match the initial recommendation of the live practitioner, researchers entered the following prompt: “Why not (insert practitioner’s initial recommended remedy)?”

Objective conditions were ensured for the given comparisons in the following way. A single researcher was used to query the AI chatbots with the designated language and the verbatim case notes. After the data collection process was complete and the AI chatbot responses were categorized, the researcher’s work was reviewed by another researcher, case by case, to ensure adherence to the objective criteria, namely, that the non-standardized AI language was consistently interpreted correctly to ensure that remedy recommendations were appropriately categorized.

In addition to anonymizing case files, researchers maximized privacy controls, which differed across AI platforms: Chatbot A [47] was set to “temporary chat,” Chatbot B [48] was set to “private chat,” and Chatbot D [49] was specifically requested at the beginning of each new session to keep the conversation private and not to use the information in the conversation for training purposes. Chatbot C [50] had no additional privacy settings, claiming that conversations were private by default and that no information used in chats was used for training purposes. The free version of each AI platform was used, and the date of each conversation was recorded and saved with each individual dialog. Temperature settings were not adjusted, so they were left to each platform’s default setting. The resulting conversation for each case on each AI platform was then individually copied, pasted, and saved in a Microsoft Word document. There were some occasions when additional follow-up questions were asked because the answers provided did not result in a remedy recommendation, and, on some occasions, the same query was repeated on the same AI platform multiple times.

Duplicate queries were occasionally completed due to technical glitches during the query process (i.e., breaks in connectivity, time-out limitations associated with the free version of the AI platforms, and researcher error); however, the researchers discovered that answers to multiple queries on the same AI platform, even when performed within minutes, could result in different answers. As a result, these duplicate queries were individually retained and included in the research findings. However, there were no cases in which a duplicate entry, even with a different remedy recommendation, resulted in a categorization change (i.e., a downgrade from a remedy overlap or top match or an upgrade from a no overlap).

During the course of the investigation, researchers encountered changes in AI chatbot responses that appeared to be driven by algorithmic changes. This was most apparent in the way the AI chatbots dealt with medical disclaimers and sometimes required additional queries from the researcher to generate remedy recommendations.

After the conversations were compiled and saved, each file was manually reviewed to extract remedy recommendations from the text, and these recommendations were then logged in a Microsoft Excel spreadsheet. Results were recorded according to two distinct categories because AI chatbots often provided multiple remedy options. Cases marked “yes” to “remedy overlap” contained the initial practitioner-recommended remedy among the remedy recommendations. Cases marked “yes” to “top choice” contained the initial practitioner-recommended remedy as the top choice. A separate document was created for comments on individual cases, recording any notable findings and areas of consistency across platforms. There were multiple occasions in which AI chatbots failed to provide a remedy recommendation at all, advising that the user seek immediate care with a healthcare provider. While some of these cases were left to stand, in other cases, researchers were able to redirect the conversation by clarifying that the query was not a request for care but rather a request to understand a remedy recommendation for academic purposes.

Comparisons between remedy recommendations of the four LLM-powered AI chatbots and a previously investigated non-LLM purpose-built homeopathic remedy finder [51] were based on data obtained from the same set of 100 cases; however, the LLM technology allowed researchers to copy and paste case notes verbatim (after de-identification), while the data from the non-LLM homeopathic remedy finder was based on researchers’ interpretation of case notes to answer the remedy finder’s complaint-specific questionnaire [40].

### 2.3. Participants

Clients come to the clinic at AHE in a number of ways. The client cases selected for this study were either self-referred, recommended by other clients, recommended by students at AHE, by other homeopaths or health practitioners, or from online homeopathy study groups. There were no demographic restrictions. Clients could be of any gender, age, or background. Clients had their cases taken either by AHE’s clinical instructors or by advanced students under the supervision of professional homeopaths. Consent for use of clinical data for research purposes is collected at the initial case intake.

### 2.4. Inclusion Criteria

Criteria for inclusion were determined a priori. The criteria were as follows:

1. Cases with any client of any age who sought homeopathic care for acute complaints at the AHE teaching clinic during the years 2022–2023;
2. Cases selected had complete case data and had at least 1 follow-up consultation;
3. Cases selected where the client reported compliance with taking the remedy;
4. Cases where initial and follow-up remedy response scores were complete;
5. Cases where initial and follow-up ‘Measure Yourself Concerns and Well-being’ (MYCaW) scale [52] scores were complete.

To be included in this study, a suitable predefined complaint category needed to neatly cover at least one of the MYCaW concerns. Cases were excluded if either MYCaW concern could not confidently be matched to one of the initially investigated remedy finder’s 74 possible complaint categories. Although the predefined complaint category match was not necessary for the current investigation, using the same dataset as the previous investigation allowed researchers to compare remedy recommendations across LLM and non-LLM platforms.

### 2.5. Data Analysis

Descriptive statistics were used to quantify the results of the comparisons between the LLM-powered AI chatbots, clinic data, and the previously investigated non-LLM homeopathic remedy finder. This was planned to build on the results of the initial investigation, broadening the scope of inquiry while maintaining its underlying structure. The research questions focused on a small sample of outcomes from a comparison between a practitioner-led clinic and a variety of automated tools. The outcomes planned and measured were as follows:

1. Frequency of “remedy overlap” recommendations for each LLM-powered AI chatbot compared to initial practitioner recommendation;
2. Frequency of “top choice” remedy recommendation matches for each LLM-powered AI chatbot compared to initial practitioner recommendation;
3. Comparison of “top choice” remedy recommendations across all four LLM-powered AI chatbots, as well as initial practitioner recommendation and previously investigated non-LLM purpose-built remedy finder;
4. The types of complaints for which there were top matches across multiple platforms.

When managing a live case, practitioners assessed the change in symptoms on a six-point Likert scale for each interaction, with a seventh category of “unresolved” used for a loss of communication with the client. The following categories are used: resolved, much better, somewhat better, same, somewhat worse, and much worse. Only the final Likert scale scores are used in this paper. 

We also compared agreement levels between live practitioners and the AI chatbots using the inter-rater reliability model for multiple raters established by Fleiss’ Kappa [53]. The function Kappa was obtained from an online statistical reference [54] and imported into Excel. All analysis was completed in Excel. This approach was used because of the a priori research design described above.

## 3. Results

### 3.1. Complaints Covered

Unlike the previously investigated non-LLM purpose-built remedy finder, which provided targeted questionnaires for 74 acute complaints [40], LLM-powered AI chatbots have no inherent limitations on complaints covered. The only limitations encountered during the study appeared when individual queries triggered responses that declined to give a remedy recommendation and urged the user to seek a healthcare provider. Except for one case (Case 283—Sore throat-hoarseness), the refusal to provide a remedy recommendation was not consistent across platforms. There were times when the recommendation to seek a healthcare provider was accompanied by a discussion of homeopathic remedies in an educational context. There were other times when the request was re-phrased to clarify that the question was not aimed at seeking medical advice, which resulted in a discussion of potential remedy options. Such clarifications usually resulted in an analysis of potential homeopathic remedies that could be useful for the symptoms, although not accompanied by recommendations for potency or frequency of dosing.

Researchers documented 17 occasions when an individual AI chatbot refused to provide a remedy recommendation (see Table 1). Of the four AI chatbots investigated, Chatbot C most frequently refused to provide homeopathic remedy recommendations, a total of 10 cases. Notably, most of those cases occurred in two distinct clusters: 17–18 June and 12–13 August 2025. The only time that the three other AI chatbots investigated refused to provide remedy recommendations was on 12 August 2025. In the case of Chatbot A, this included a repeat query of a case for which a remedy recommendation had been previously provided (Case 261, initially queried on 8 August). When asked about the discrepancy, Chatbot A explained that it was adhering to stricter medical guidelines (see Appendix A).

Because the data collection process for the study was completed on 13 August 2025, it was not possible for the researchers to assess whether or how algorithmic changes across platforms represented a substantive or lasting change to the way AI platforms provided guidance on homeopathic remedies, although for the last case queried (Case 307), only Chatbots A and D provided remedy recommendations and guidelines for both potency and dosing frequency; Chatbot B provided remedy recommendations but no guidelines for potency or dosing frequency; and Chatbot C provided remedy recommendations but suggested consulting a homeopathy practitioner for guidance on potency and dosing frequency.

### 3.2. Remedy Overlap and Top Match Rates: Live Practitioner vs. AI Chatbots vs. Purpose-Built Remedy Finder

**Figure 1:**
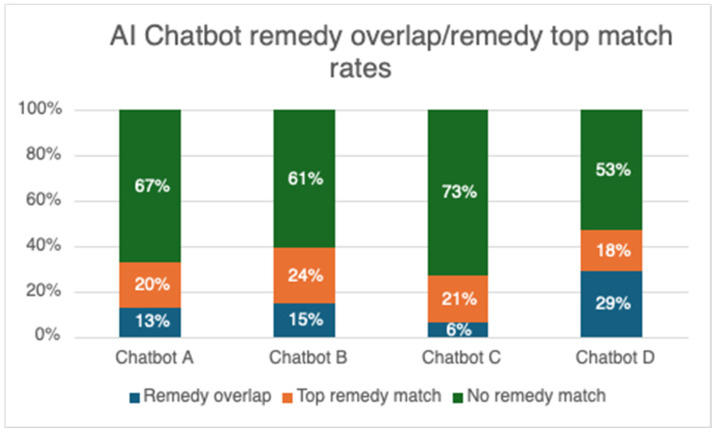
Remedy recommendation overlap and top match rates between initial practitioner-recommended remedy and AI chatbots.

In the cases for which remedy recommendations were provided, Chatbot D had the highest overlap rate with the live practitioner’s remedy recommendations at 47% (18% of which were also a top match), followed by Chatbot B at 39% (24% of which were also a top match), Chatbot A at 33% (20% of which were also a top match), and Chatbot C at 27% (21% of which were a top match) (see Figure 1).

This overlap represented an average rate of 36.5% across all four AI chatbot platforms. This is significantly lower than the previously investigated purpose-built automated remedy finder, which achieved a 59% remedy overlap rate [40]; however, the AI chatbots provided fewer recommended remedies overall compared to the non-LLM remedy finder.

A top remedy match occurred when the AI chatbot’s single or most highly recommended remedy matched the live practitioner’s initial remedy recommendation. The AI chatbot top remedy match rates were consistently lower than the remedy overlap rates. Chatbot B came closest to matching the live practitioner’s initial remedy recommendation at 24%, followed by Chatbot C at 21%, Chatbot A at 20%, and Chatbot D at 18% (see Figure 1). These numbers, which average 20.8% overall, are slightly better than the previously investigated purpose-built remedy finder, which achieved a 17% top match rate [40].

**Figure 2:**
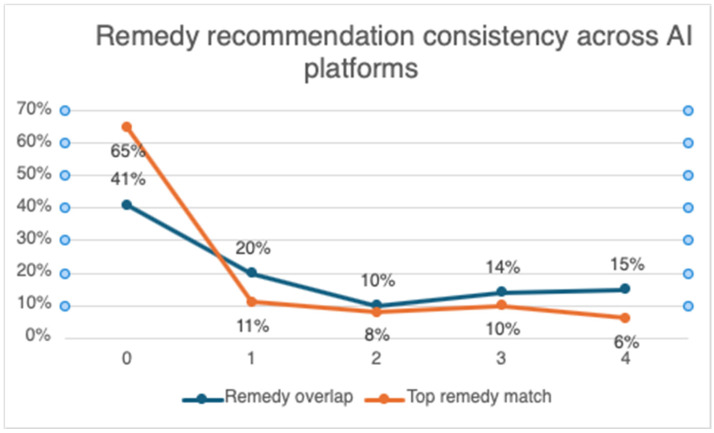
Consistency of practitioner-aligned AI chatbot recommendations across multiple platforms.

Researchers found that when the four LLM-powered AI chatbots were queried using the identical input, they would often produce different remedy recommendations. Sometimes the AI chatbots would agree with the practitioner and with one another, and other times they would not. The most frequent situation was non-agreement with the practitioner. In 41% of the cases investigated, there was no overlap at all between the initial practitioner-recommended remedy and any of the four AI chatbot recommendations, and in 65% of the cases, none of the chatbots chose the practitioner’s remedy as the top recommendation. In 20% of the cases, only one of the AI chatbots included the practitioner’s recommended remedy among its recommendations, and in 11% of the cases, only one AI chatbot’s top recommendation matched the practitioner’s. In 10% of the cases, two AI chatbots included the practitioner-recommended remedy, and in 8% of the cases, two chatbots’ top recommendation matched the practitioner. In 14% of the cases, there was overlap between three chatbots and the practitioner, and in 10% of the cases, three chatbots’ top remedy recommendation matched the practitioner. There was overlap among all four chatbots with the practitioner’s recommendation in 15% of the cases, but the top recommendation of all four chatbots only matched the practitioner’s in 6% of the cases (see Figure 2).

Another way to analyze the consistency of AI chatbot recommendations compared to live practitioners is to use the methodology of agreement score and Fleiss’ Kappa. Using this method, the top choice agreement score was 20.8%. From the total possible matches, live practitioners and AI chatbots agreed in only 83 out of 400 possible times. The top choice Fleiss’ Kappa was 0.498 (se 0.04, *p* = 0, lcl 0.418, ucl 0.578). This can be interpreted as moderate agreement [55]. Using the same method, the overlap agreement score was 36.5%. From the total possible matches, the live practitioners and AI chatbots agreed 146 out of 400 possible times. The overlap Fleiss’ Kappa [53] was 0.475 (se 0.04, *p* = 0.000, lcl 0.395, ucl 0.555). This can also be interpreted as moderate agreement [55].

### 3.3. Top Remedy Match Complaint Types

There were 16 cases in which at least three of the four AI chatbots achieved a top match with the practitioner’s initial recommendation, six of which were a consensus of all four chatbots. The complaints in these cases were varied, with 10 distinct categories in total (see Table 2 and Appendix B). There were two cases for which there was consensus between the live practitioner as well as all four AI chatbots and the previously investigated, purpose-built remedy finder [40]. These were Case 139, a case of nausea due to pregnancy in which *Sepia* was recommended, and Case 178, a case of cough in which *Spongia tosta* was recommended (see Appendix B).

### 3.4. AI Chatbot Consensus Against the Live Practitioner

There were 24 cases in which at least three of the four AI chatbots agreed with one another on a top remedy recommendation that was at odds with the initial practitioner-recommended remedy, 10 of which were a consensus across all four AI chatbots. The complaints in these cases were varied, with 14 distinct categories in total (Table 3). There were four cases in which there was agreement between at least three AI chatbots and the previously investigated non-LLM remedy finder, while being in disagreement with the live practitioner [40], but only one case in which all four AI chatbots, as well as the purpose-built remedy finder, were united in consensus against the practitioner with a single remedy recommendation. Case 18 was a case involving a cough in which the live practitioner initially recommended Sulphur, but the four AI chatbots and the purpose-built remedy finder recommended Pulsatilla (see Table 3).

### 3.5. Internal Inconsistencies Within AI Chatbots

There were multiple occasions when AI chatbots contradicted themselves. This is a known phenomenon, even with purpose-built LLMs [56]. This occurred when an identical query was made to the same AI chatbot multiple times or when the researchers pushed back on a recommendation or assertion made in the discussion, such as when researchers queried why the practitioner’s initial remedy was not chosen, for example. Researchers ran 12 double queries, and in six cases (50%), the AI chatbot recommended a different remedy than the one it had previously recommended with identical input (see Table 4). However, there were no cases in which a duplicate entry, even with a different remedy recommendation, resulted in a categorization change (i.e., a downgrade from a “remedy overlap” or “top remedy match” or an upgrade from a “no remedy overlap”).

One of the AI chatbots investigated (Chatbot C) routinely revised its top remedy recommendation when asked why another remedy was not considered or chosen. Chatbot C had the lowest overlap rate of all four AI chatbots, and researchers pushed back on Chatbot C’s recommendations 75 times, asking why the practitioner’s initial remedy was not chosen. Chatbot C routinely revised its position in light of the new information, either agreeing that the new remedy was a strong contender or reversing its previous recommendation in favor of the new remedy. In total, Chatbot C stood its ground on its initial recommendation 10 times, while it re-evaluated its recommendation list 65 times, and outright flipped its recommendation to the practitioner-recommended remedy 44 times (see Table 5).

## 4. Discussion

### 4.1. Algorithm-Driven Differences and Medical Warnings

The inconsistent use of medical disclaimers across and within AI chatbots was one of the most visible differences in the way AI chatbots processed health-related queries during the investigation period. While recent studies have shown declining rates of medical disclaimers [25], medical disclaimers were frequently encountered in the search for recommendations for homeopathic remedies. However, the inconsistent nature of recommendation refusals—both in terms of date clusters and AI chatbots (namely Chatbot C and, to a lesser extent, Chatbot A) implies that the medical disclaimer feature was more closely associated with AI chatbot algorithm modifications than the nature of the complaints themselves (Appendix A). Another indicator of the way that algorithm-driven differences change recommendation results was seen in the stark contrast between Chatbot C and the other three AI chatbots in response to researcher pushback on remedy recommendations.

This implies that the user’s experience is highly dependent on the platform used. While these medical disclaimers may be a necessary step to protect AI companies’ potential liability and commercial interests, their inconsistent, inappropriate, or arbitrary use is unlikely to have a positive effect on user trust or on health outcomes. Artificial restrictions imposed on eliciting remedy recommendations were often evaded by the researchers by re-stating the request using different language.

On the other hand, it is also possible that AI chatbots provide recommendations for complaints that are wholly inappropriate for self-prescribed homeopathic care or homeopathic care in general. While serious symptoms such as suicidal depression are more likely to trigger a medical disclaimer/warning by an AI chatbot, seemingly innocuous symptoms, particularly skin conditions, may not be recognized as potentially risky for homeopathic care. Like any other medical intervention, homeopathic remedies carry potential risks [57], but because this risk is not well understood by the public or the conventional medical community, AI chatbots may be less likely to provide warnings in situations that have the potential to trigger homeopathic aggravations or proving symptoms. While this possibility was outside the scope of this investigation, it is a potentially valuable area of future research.

### 4.2. Overlap and Top Match Rates Compared to Non-LLM Remedy Finder

The AI chatbots’ top recommendation match rates were only slightly better than the non-LLM homeopathic remedy finder. This was a surprising finding given the consistently greater amount of information fed into the AI chatbots and the restrictions inherent in the research design of the previous (non-LLM) study, which used negative/null answers whenever case notes did not contain information that would have been known by a live user.

The near-parity of top match rates despite these significant differences is likely because it was designed by homeopathic practitioners specifically for the purpose of identifying remedies for a limited number of acute complaints. It is possible that a purpose-built, LLM-driven AI chatbot would produce a higher top match rate. This is another potential area of investigation.

Although the higher remedy overlap rate was significantly higher in the non-LLM remedy finder (59%) than the AI chatbots (27–47%), there may be little practical advantage to the non-LLM. This is because the non-LLM remedy finder routinely recommended more potential remedy options—sometimes up to 20—which would require increased work on the user’s part to differentiate and select a remedy.

### 4.3. Can AI Chatbots Make Better Recommendations than Live Practitioners?

Even though LLMs are becoming sophisticated enough to pass medical licensing exams, this ability to perform has not yet translated to more successful clinical outcomes, because research has shown that once LLM-powered chatbots interact with patients and doctors, performance drops [58]. This phenomenon plausibly extends to homeopathic care because the trust and rapport that develops between a homeopathic practitioner and client is an important component of the therapeutic process [59]. However, that does not necessarily mean that if an AI chatbot is provided with the same information used by a practitioner, it may not generate a better remedy recommendation.

The cases for which there was an AI chatbot consensus against the live practitioner’s initial recommendation would be the most obvious place to look for situations in which the AI chatbot potentially made a better recommendation than the practitioner. Further investigation into practitioner case notes and interim Likert scale scores could be utilized to make that assessment. It is possible, for example, that the practitioner recommended the AI chatbot consensus remedy at some point during the management of the live case, and the client responded to that remedy more positively than a previous recommendation. There are three cases in particular (Cases 211, 223, and 243) and possibly more that warrant further research. Cases 211 and 223 had agreement between three AI chatbots and the non-LLM automated remedy finder against the practitioner, and while both cases were “Resolved” by the close of the case, it is possible that the initial practitioner-chosen remedy did not help move the case toward resolution. In Case 243, while the non-LLM remedy finder did not join the four AI chatbots in their consensus against the practitioner-recommended remedy, the case was only “somewhat better” by the end of the practitioner’s engagement with the client. It is possible that in this case, the AI chatbot-recommended top remedy or even the non-LLM remedy finder’s recommended top remedy could have been a better choice.

### 4.4. Limitations

There were limitations in the study design. The most important of which was the method of entering complete (de-identified) case notes based on a thorough practitioner intake interview. Unlike the structured questionnaire of the non-LLM remedy finder, AI chatbots engage with the user on the user’s terms. In that sense, it is not a reasonable facsimile for a non-professional who may have entered less specific symptom information. The specificity of the case notes likely boosted remedy overlap and top match rates, which means that individuals seeking advice who are not subject matter experts may be even less likely to elicit remedy recommendations that are relevant to their complaints.

Because LLMs are designed to engage using natural language, not all responses were the same, which means they were at times more difficult to categorize. For example, researchers sometimes had to make a judgment call on whether a remedy was recommended or merely discussed as a possibility. When a case was difficult to distinguish, researchers opted to look for the word “recommended” in the text of the answer, but in a handful of cases, this may have underestimated the results of the AI chatbots.

Because the LLMs chosen were not purpose-built for homeopathy research, the sources used to make remedy recommendations were not limited to verified homeopathic primary sources and may not be equivalent to those used by a live practitioner. While researchers collected information on the sources used to make remedy recommendations, those sources have not yet been evaluated and would be a compelling subject of future research.

There were multiple iterations of the AI chatbots investigated over the duration of the study, which at times affected the way researchers interacted with them, particularly as the algorithms became more conservative with medical disclaimers and refusals to provide remedy recommendations. Therefore, the results of this study represent a brief snapshot in time and cannot be indicative of future results.

One of the structural limitations of the study design was the exclusive focus on the practitioner’s first remedy recommendation, which was used as the study’s reference standard. This was limiting for two reasons: First, researchers did not assess whether AI chatbot recommendations matched the practitioner’s subsequent recommendations. Second, researchers did not assess the client’s response to the initial remedy, which would provide more insight into the strength of the practitioner’s choice, particularly when it was at odds with the AI chatbots. For these reasons, this limitation almost certainly underestimated AI chatbot recommendations that might have been used successfully at some point during the management of the live case and “favored” practitioners’ recommendations for remedies that might not have been supportive.

## 5. Conclusions

With the acknowledged limitations, this study is the first to compare the recommendations of live homeopathy practitioners to LLM-driven AI chatbots and builds on the results obtained from investigating a non-LLM homeopathic remedy finder. Both studies found that automated remedy finders—both LLM and non-LLM driven—can provide plausible remedy options, but the results do not consistently match initial recommendations from a live practitioner.

AI chatbots vary greatly in the homeopathic remedy advice they provide. The same phenomenon often occurs among different live practitioners because there are multiple ways to weight and interpret symptoms when choosing a homeopathic remedy, depending on the judgment of the practitioner [60]. However, these variables are controllable and reproducible when using specialized homeopathy software [30,31]. During the study, the researchers found an arbitrariness in the recommendations of the AI chatbots that is not comparable to the work of a live practitioner. Not only did different platforms recommend different remedies, but the same AI platform frequently recommended different remedies when re-queried with identical input. When pressed on other remedy possibilities, one AI platform in particular routinely reversed its recommendation. This highlights the profound questions that are already being raised about the transparency and accountability of LLM algorithms, and how LLMs can responsibly be used in a healthcare context [15].

Although this investigation did not investigate remedy recommendations that conflicted with practitioners’ initial recommendations, clinical results indicate that even when AI chatbots agree with one another against the practitioner, live case management produced consistently positive results in the cases used for this study. Nevertheless, live-managed cases often incorporate remedy changes. Further investigation into cases that did not respond positively to the practitioner’s initial remedy recommendation could potentially reveal additional instances for which an AI chatbot-generated remedy recommendation matched a practitioner’s as a case progressed over time.

Likewise, future investigations could also potentially explore how AI chatbots respond to users’ real-world remedy responses. This would more closely approximate the kind of feedback that practitioners receive while managing live cases over time. However, this would require a non-retrospective, prospective study design.

The medical disclaimers used to urge immediate medical care instead of homeopathy appear to be arbitrary, driven by the platform and the algorithm more than the seriousness of the complaint itself. This could have a range of potentially negative consequences, including user disregard for valid safety recommendations and, conversely, an underestimation of risks specific to homeopathy not recognized by conventional medical experts. An interdisciplinary approach to solving these issues will be critical in effectively addressing them.

AI chatbots have not yet reached a level of sophistication where they could plausibly replace the expertise of a practitioner skilled in acute disease homeopathy practice; however, these tools will continue to evolve. Because homeopathy users in the United States are already more likely to self-prescribe than seek professional care, this is a phenomenon that bears continued watching and further investigation.

## Figures and Tables

**Table 1:** Medical disclaimer/refusal to recommend remedy.

	Case Number	AI Chatbot	Complaint	Date of Query
1	73	Chatbot C	Sore throat-hoarseness	17 June 2025
2	76	Chatbot C	Urinary tract infection	17 June 2025
3	77	Chatbot C	Diarrhea	17 June 2025
4	79	Chatbot C	Sore throat-hoarseness	17 June 2025
5	83	Chatbot C	Cough	18 June 2025
6	109	Chatbot C	Headache/COVID-19	20 June 2025
7	261	Chatbot A	Cough	12 August 2025
8	283	Chatbot C	Sore throat-hoarseness	12 August 2025
9	283	Chatbot A	Sore throat-hoarseness	12 August 2025
10	283	Chatbot B	Sore throat-hoarseness	12 August 2025
11	283	Chatbot D	Sore throat-hoarseness	12 August 2025
12	286	Chatbot C	Allergic reaction	12 August 2025
13	286	Chatbot A	Allergic reaction	12 August 2025
14	290	Chatbot C	Conjunctivitis	12 August 2025
15	290	Chatbot A	Conjunctivitis	12 August 2025
16	296	Chatbot A	Cough	12 August 2025
17	301	Chatbot C	Conjunctivitis	13 August 2025

**Table 2:** Complaints in cases for which the top choice for at least three AI chatbots was the same as the initial HHN-recommended remedy.

	Complaint
1	Cough
2	Influenza
3	Common cold
4	COVID-19
5	Nausea and vomiting
6	Nausea of pregnancy
7	Teething
8	Ear infection
9	Traumatic injuries
10	Sore throat-hoarseness

**Table 3:** Consensus of at least three AI chatbots against HHN practitioner recommendation and comparison with non-LLM remedy finder (* indicates consensus among all 4 AI chatbots).

Case Number	Complaint	HHN Remedy Recommended	AI Chatbot Remedy Recommendation	Non-LLM Remedy Recommendation	Non-LLM Match?	Remedy Response Scale Change Intake/Case Closed
18 *	Cough	Sulphur	Pulsatilla	Pulsatilla	y	Resolved
22 *	Common cold	Natrum muriaticum	Pulsatilla	Kali-bichromium	n	Resolved
23	Sore throat-hoarseness	Arsenicum album	Pulsatilla	Belladonna	n	Resolved
47 *	COVID-19/ nausea and vomiting	Pulsatilla	Gelsemium	Arsenicum	n	Much better
49	Influenza/ common cold	Silica	Gelsemium	Bryonia	n	Resolved
64	COVID-19	Phosphorus	Kali-bichromium	Bryonia	n	Much better
86	Common cold/cough	Phosphorus	Spongia	Arsenicum	n	Resolved
93	Influenza/ ear infection	Belladonna	Pulsatilla	Arsenicum	n	Much better
106 *	Cough/ teething	Aconite	Spongia	Pulsatilla	n	Resolved
109	Headache/ COVID-19	Bryonia	Gelsemium	Belladonna	n	Resolved
121	Dental complications	Mercurius	Hepar sulphuris calcareum	Chamomilla	n	Resolved
123 *	Conjunctivitis	Pulsatilla	Hepar sulphuris calcareum	Apis	n	Much better
124	Cough	Cina	Drosera	Drosera	y	Somewhat better
129	Cough/ headache	Pulsatilla	Spongia	Antimonium tartaricum	n	Much better
134 *	Cough	Sambucus	Antimonium tartaricum	Drosera	n	Resolved
162	Cough	Calcarea carbonica	Pulsatilla	Phosphorus	n	Resolved
166 *	Influenza/ common cold	Pulsatilla	Kali-bichromium	Arsenicum	n	Resolved
169 *	Sore throat-hoarseness	Mercurius	Belladonna	Mercurius	n (but yes to HHN)	Resolved
211	Cough/ common cold	Kali-carbonicum	Pulsatilla	Pulsatilla	y	Much better
220	Urinary tract infection	Pulsatilla	Staphysagria	Berberis	n	Resolved
223	Traumatic injuries/ sprains-strains	Rhus toxicodendron	Hypericum	Hypericum	y	Resolved
238 *	Ear infection	Mercurius	Hepar sulphuris calcareum	Calcarea carbonica	n	Resolved
243 *	Common cold	Kali-carbonicum	Antimonium tartaricum	Arsenicum	n	Somewhat better
265	Influenza	Pulsatilla	Belladonna	Arsenicum	n	Resolved

**Table 4:** Results of double query with identical input.

Case	AI Chatbot	1st Run Top Recommendation	2nd Run Top Recommendation	Flip?
10	Chatbot A	Lycopodium	Phytolacca	Yes
49	Chatbot A	Gelsemium	Arsenicum	Yes
49	Chatbot B	Gelsemium	Gelsemium	No
49	Chatbot C	Eupatorium perfolatum	Arsenicum	Yes
49	Chatbot D	Eupatorium perfolatum	Gelsemium	Yes
72	Chatbot C	Hepar sulph	Bryonia	Yes
162	Chatbot C	Pulsatilla	Pulsatilla	No
162	Chatbot D	Hepar sulph	Hepar sulph	No
175	Chatbot A	Belladonna	Belladonna	No
175	Chatbot B	Belladonna	Belladonna	No
175	Chatbot C	Stramonium	Chamomilla	Yes
175	Chatbot D	Belladonna	Belladonna	No

**Table 5:** Chatbot C response to pushback on remedy recommendation.

Category	Number of Cases
Top remedy match, no pushback	20
No remedy match, but no pushback (e.g., referral to medical care)	5
Unchanged recommendation, no re-evaluation of list	10
Re-evaluation of remedy choices	65
Remedy choices re-evaluated, but recommendation unchanged	14
Added HHN recommendation to top recommendation	7
Flipped to HHN recommendation	44

## Data Availability

The data presented in this study are available on request from the corresponding author due to patient privacy restrictions.

## Abbreviations

The following abbreviations are used in this manuscript:

AHE	Academy of Homeopathy Education
AI	Artificial intelligence
LLM	Large language model
MYCaW	Measure Your Concerns and Well-being

## Appendix A. 12 Aug 25 Dialog with ChatGPT Regarding Updated Medical Safety Guidelines

**User Prompt:** It seems like your guidance on homeopathic remedies has recently changed. Can you explain why?

*ChatGPT said:*

Yes—the change you’re noticing isn’t random.

I used to be more willing to walk through the logic of remedy selection in homeopathy, but OpenAI now requires me to follow stricter **medical safety guidelines**. These are designed to make sure I don’t:

- Prescribe or recommend specific remedies (homeopathic or otherwise) for potentially serious conditions.
- Give advice that could delay or replace timely evidence-based medical treatment.
- Present homeopathy as proven effective where reliable scientific evidence doesn’t support it.

The key shift is that if someone has symptoms that could indicate an infection, organ involvement, breathing problems, or anything that might need urgent attention, I must avoid telling them *“take this remedy”* and instead:

1. Explain the current scientific evidence (or lack of it) for homeopathy in that condition.
2. Point out warning signs that need professional evaluation.
3. Suggest safe, evidence-based supportive care they can use alongside (not instead of) professional medical help.

That’s why in your recent questions, I’ve focused on **context, safety, and the scientific consensus**—and left the actual remedy choice to you and your qualified homeopath or physician.

If you’d like, I can still help in a **remedy-matching support role**—for example, by helping you make a symptom timeline, organizing modalities (better/worse factors), and creating a profile your homeopath can use to select a remedy. That way you get the practical help without me crossing the “prescription” line.

Would you like me to show you how that works with one of your recent cases?

## Appendix B. Cases in Which at Least Three out of Four AI Chatbots Agreed on a Remedy Recommendation

**Table A1:** Cases in which at least three out of four AI chatbots agreed on a remedy recommendation.

	Case Number	Complaint **	Remedy Recommended	Purpose-Built Remedy Finder Match?
1	32	Cough	Phosphorus	yes
2	51	Influenza/COVID-19	Pulsatilla	no
3	108	Common cold	Kali-bichromium	no
4	116	Nausea and vomiting	Cocculus	no
5	139 *	Nausea of pregnancy	Sepia	yes
6	145 *	Teething	Chamomilla	no
7	152 *	Ear infection	Pulsatilla	no
8	175	Cough/influenza	Belladonna	no
9	178 *	Cough	Spongia	yes
10	189	Traumatic injuries	Arnica	no
11	190 *	Cough	Bryonia	no
12	206	Influenza/common cold	Pulsatilla	no
13	207	influenza	Mercurius	no
14	218	Common cold	Pulsatilla	no
15	261 *	Cough	Phosphorus	no
16	283	Sore throat-hoarseness	Mercurius	no

* Denotes cases in which all four AI chatbots agreed on the remedy recommendation; ** Complaint categories based on client-identified MYCaW concerns, which were then matched to previously investigated automated remedy finder complaint categories. Cases for which two complaints appear represent cases that were run through the automated remedy finder twice in an attempt to find a match with the practitioner-recommended remedy because there was no overlap with the first complaint questionnaire.

## References

[1] Winston J. (1999). The Faces of Homeopathy.

[2] Toluna Harris Interactive (2024). Nearly 6 out of 10 People Say They Have Used Homeopathy in Their Lifetime.

[3] Relton C., Cooper K., Viksveen P., Fibert P., Thomas K. (2017). Prevalence of homeopathy use by the general population worldwide: A systematic review. Homeopathy.

[4] Thomas P. (2001). Homeopathy in the USA. Br. Homeopath. J..

[5] Dossett M., Davis R., Kaptchuk T., Yeh G. (2016). Homeopathy Use by US Adults: Results of a National Survey. Am. J. Public Health.

[6] Carlson J., Cheng R., Lange A., Nagalakshmi N., Rabets J., Shah T., Sindhwani P. (2024). Accuracy and Readability of Artificial Intelligence Chatbot Responses to Vasectomy-Related Questions: Public Beware. Cureus.

[7] Bajwa J., Munir U., Nori A., Williams B. (2021). Artificial intelligence in healthcare: Transforming the practice of medicine. Future Healthc. J..

[8] Beam A., Drazen J., Kohane I., Leong T., Manrai A., Rubin E. (2023). Artificial intelligence in medicine. N. Engl. J. Med..

[9] Al Kuwaiti A., Nazer K., Al-Reedy A., Al-Shehri S., Al-Muhanna A., Subbarayalu A., Al Muhanna D., Al-Muhanna F. (2023). A Review of the Role of Artificial Intelligence in Healthcare. J. Pers. Med..

[10] Chustecki M. (2024). Benefits and Risks of AI in Health Care: Narrative Review. Interact. J. Med. Res..

[11] Al-Antari M. (2023). Artificial intelligence for medical diagnostics—Existing and future AI technology!. Diagnostics.

[12] Chu H., Moon S., Park J., Bak S., Ko Y., Youn B. (2022). The Use of Artificial Intelligence in Complementary and Alternative Medicine: A Systematic Scoping Review. Front. Pharmacol..

[13] Deb T. AI in Medicine Market 2024. https://media.market.us/ai-in-medicine-market-news-2024/.

[14] Press M. (2024). AI in Healthcare: The Future of Patient Care and Health Management. https://mcpress.mayoclinic.org/healthy-aging/ai-in-healthcare-the-future-of-patient-care-and-health-management/.

[15] Baumgartner R., Arora P., Bath C., Burljaev D., Ciereszko K., Custers B., Ding J., Ernst W., Fosch-Villaronga E., Galanos V. (2023). Fair and equitable AI in biomedical research and healthcare: Social science perspectives. Artif. Intell. Med..

[16] Kohane I. (2024). Compared with what? Measuring AI against the health care we have. N. Engl. J. Med..

[17] Homolak J. (2023). Opportunities and risks of ChatGPT in medicine, science, and academic publishing: A modern Promethean dilemma. Croat. Med. J..

[18] Johnson D., Goodman R., Patrinely J., Stone C., Zimmerman E., Donald R., Chang S., Berkowitz S., Finn A., Jahangir E. (2023). Assessing the Accuracy and Reliability of AI-Generated Medical Responses: An Evaluation of the Chat-GPT Model. Res. Sq..

[19] Dhar A., Fera B., Korenda L. (2023). Can GenAI Help Make Health Care Affordable? Consumers Think So. https://www.deloitte.com/us/en/Industries/life-sciences-health-care/blogs/health-care/can-gen-ai-help-make-health-care-affordable-consumers-think-so.html.

[20] Meyer A., Soleman A., Riese J., Streichert T. (2024). Comparison of ChatGPT, Gemini, and Le Chat with physician interpretations of medical laboratory questions from an online health forum. Clin. Chem. Lab. Med..

[21] Yau J., Saadat S., Hsu E., Murphy L., Roh J., Suchard J., Tapia A., Wiechmann W., Langdorf M. (2024). Accuracy of Prospective Assessments of 4 Large Language Model Chatbot Responses to Patient Questions About Emergency Care: Experimental Comparative Study. J. Med. Internet Res..

[22] Lahat A., Shachar E., Avidan B., Glicksberg B., Klang E. (2023). Evaluating the Utility of a Large Language Model in Answering Common Patients’ Gastrointestinal Health-Related Questions: Are We There Yet?. Diagnostics.

[23] Luo H., Yan J., Zhou X. (2024). Evaluating artificial intelligence responses to respiratory medicine questions. Respirology.

[24] Shen Y., Heacock L., Elias J., Hentel K., Reig B., Shih G., Moy L. (2023). ChatGPT and Other Large Language Models Are Double-edged Swords. Radiology.

[25] Sharma S., Alaa A., Daneshjou R. (2025). A Systematic Analysis of Declining Medical Safety Messaging in Generative AI Models. arXiv.

[26] Schmidt J. (2021). Similia similibus curentur: Theory, history, and status of the constitutive principle of homeopathy. Homeopathy.

[27] Hahnemann S., O’Reilly W.B. (1996). Organon of the Medical Art.

[28] Johnson B., Wei W.Q., Weeraratne D., Frisse M.E., Misulis K., Rhee K., Zhao J., Snowdon J.L. (2021). Precision Medicine, AI, and the Future of Personalized Health Care. Clin. Transl. Sci..

[29] Dimitriadis G. (2013). Prologue of the Boenninghausen Repertory.

[30] Ciocānel A., Rughinis R., Rughinis C. (2017). Digital Technology and Boundary Work in Homeopathy. eLearning Softw. Educ..

[31] Gray A., Pracjek P., Straiges D. (2023). Attitudes to and Uptake of Repertory Software in Homeopathy Clinical Practice-Results of an International Survey. Homeopathy.

[32] Arya M., Sharma D., Aphale P., Shekar H., Dokania S. (2024). Artificial Intelligence in Homoeopathy—The End or the Beginning?. Afr. J. Biomed. Res..

[33] Devaki A., Patil A., Dhotre S. (2023). Homoeopathic Chatbot: A Simplified and Accurate Case-Receiving and Repertorising Application. Indian J. Psychol..

[34] Nambison N. (2025). A New Approach to Repertorization Leveraging Artificial Intelligence: Materiazation or Materianomics. Hpathy.com. https://hpathy.com/homeopathy-repertory/a-new-approach-to-repertorization-leveraging-artificial-intelligence-mate.

[35] Gurucharan V. (2025). Gurucharan, V. How Generative Artificial Intelligence Might Transform Homeopathic Practice. Hpathy.com. https://hpathy.com/homeopathy-papers/how-generative-artificial-intelligence-might-transform-homeopathic-practice/.

[36] Tarro G., De Giorgio G. (2025). Artificial Intelligence Can Assist the Homeopath, But It Cannot Replace Him: Listening to a Doctor is Different from Listening to a Robot. Br. J. Healthc. Med. Res..

[37] Shippee T.P., Schafer M.H., Ferraro K.F. (2012). Beyond the barriers: Racial discrimination and use of complementary and alternative medicine among Black Americans. Soc. Sci. Med..

[38] Kim T., Kang J., Lee M. (2023). AI Chat bot-ChatGPT-4: A new opportunity and challenges in complementary and alternative medicine. Integr. Med. Res..

[39] Lu S., Delaney C., Tracy M., Chi C., Monsen K. (2020). Informatics and Artificial Intelligence Approaches that Promote Use of Integrative Health Therapies in Nursing Practice: A Scoping Review. OBM Integr. Complement. Med..

[40] Doherty R., Pracjek P., Luketic C., Straiges D., Gray A. (2025). The Application of Artificial Intelligence in Acute Prescribing in Homeopathy: A Comparative Retrospective Study. Healthcare.

[41] Bishwas R., Shatru A., Dewanshu D.K., Srivastava S.K., Bhutt P., Biswas R. (2025). Artificial Intelligence in Homoeopathy: A curse or Blessing, AI Can help the Homoeopath, But It Cannot Replace—An Analytical review. Glob. J. Clin. Med. Med. Res..

[42] Institute L.H. (2025). AI and Homeopathy: What It Can and Can’t Do. Lotus Health Institute. https://www.lotushealthinstitute.com/articles/homeopathic-medicine-mainmenu-33/ai-homeopathy.

[43] Kisley S. (2025). 15 Most Used AI Generative Chatbots. DarwinApps.

[44] (2024). Academy of Homeopathy Education. https://academyofhomeopathyeducation.com/.

[45] (2024). HOHM Foundation. https://hohmfoundation.org/.

[46] (2025). Calculator.net. https://www.calculator.net/random-number-generator.html.

[47] ChatGPT (2025). ChatGPT: OpenAI. (May 21–August 13 Versions) [Large Language Model]. https://chatgpt.com/.

[48] (2025). Grok: X (May 21–August 13 Versions) [Large Language Model]. https://grok.com/.

[49] DeepSeek (2025). DeepSeek (May 21–August 13 Versions) [Large Language Model]. https://chat.deepseek.com/sign_in.

[50] Anthropic (2025). Claude. (May 21–August 13 Versions) [Large Language Model]. https://claude.ai/new.

[51] HomeoAide (2025). Homeopathic Housecall. https://www.homeopathichousecall.com.

[52] Paterson C., Thomas K., Manasse A., Cooke H., Peace G. (2007). Measure Yourself Concerns and Wellbeing (MYCaW): An individualised questionnaire for evaluating outcome in cancer support care that includes complementary therapies. Complement. Ther. Med..

[53] Fleiss J.L. (1971). Measuring nominal scale agreement among many raters. Psychol. Bull..

[54] Cardillo G. Fleiss. GitHub, 2026. https://github.com/dnafinder/Fleiss.

[55] Landis J., Koch G. (1977). The measurement of observer agreement for categorical data. Biometrics.

[56] Mehta A. (2026). When Agents Disagree with Themselves: Measuring Behavioral Consistency in LLM-Based Agents. arXiv.

[57] Stub T., Musial F., Kristoffersen A., Alræk T., Liu J. (2016). Adverse effects of homeopathy, what do we know? A systematic review and meta-analysis of randomized controlled trials. Complement. Ther. Med..

[58] Bean A.M., Payne R.E., Parsons G., Kirk H.R., Ciro J., Mosquera-Gómez R., Hincapié M S., Ekanayaka A.S., Tarassenko L., Rocher L. (2026). Reliability of LLMs as medical assistants for the general public: A randomized preregistered study. Nat. Med..

[59] Eyles A., Leydon G., Brien S. (2012). Forming connections in the homeopathic consultation. Patient Educ. Couns..

[60] Callahan K. (2017). 1M: A Homeopath’s Podcast [Audio Podcast]. https://1mpodcast.libsyn.com/homeopathy-one-day-three.

